# A genetic strategy to allow detection of F-actin by phalloidin staining in diverse fungi

**DOI:** 10.1128/msphere.00517-25

**Published:** 2025-09-29

**Authors:** Alison C. E. Wirshing, Cristina Colino-Palomino, Analeigha V. Colarusso, Mario Pinar, Daniel J. Lew

**Affiliations:** 1 Department of Biology, Massachusetts Institute of Technology 311383, Cambridge, Massachusetts, USA; 2 Department of Molecular and Cellular Biosciences, CSIC Centro de Investigaciones Biológicas Margarita Salas 383204, Madrid, Spain; NC State University, Raleigh, North Carolina, USA

**Keywords:** actin, filamentous fungi, phalloidin

## Abstract

Actin is highly conserved across eukaryotes. This versatile protein builds cytoskeletal networks central to diverse cellular processes, including cell division and cell motility. The most potent and broadly used reagents to detect polymerized actin distribution in fixed cells are fluorescently conjugated derivatives of the basidiomycete-derived toxin, phalloidin. However, despite its conservation, actin in many ascomycete fungi fails to bind phalloidin. Here, we trace the failure to bind phalloidin to a single amino acid change in a phalloidin-binding residue in actin. Reverting this change in the fungi *Aureobasidium pullulans* and *Aspergillus nidulans* by introducing the point mutation *act1^V75I^* at the native *ACT1* locus confers phalloidin binding while retaining actin function. This strategy should enable characterization of F-actin in a wider range of fungi.

**IMPORTANCE:**

High-resolution tools to visualize filamentous actin networks are critical to the investigation of organisms’ cell biology. The gold standard tool is fluorescent phalloidin, a mushroom toxin. However, several fungi have actin that fails to stain with phalloidin. Here, we describe a way to reverse that failure, rendering the invisible actin visible.

## INTRODUCTION

Actin networks drive diverse fundamental cellular processes including cell motility, cell shape, vesicle traffic, endocytosis, organelle positioning, and cytokinesis (1). The molecular mechanisms by which actin cytoskeletal networks are assembled have been elucidated, in part, through studies in the tractable yeast model systems *Saccharomyces cerevisiae* and *Schizosaccharomyces pombe* (2–4). This work has established several core functions of the actin cytoskeleton in fungi, but these simple, small yeasts do not engage in the more elaborate forms of fungal morphogenesis like building mycelial networks and fruiting bodies. Thus, studies of broader fungal morphological and biological diversity exploit other species like *Aspergillus nidulans*, whose long hyphae and genetic amenability provided a model for investigating mechanisms of long-range intracellular transport (5), and *Magnaporthe* sp., whose appressorium served as a model for the highly pressurized structures used by fungi to penetrate plant hosts (6). New genome modification technologies have accelerated the process of making non-model organisms tractable for molecular and cellular biology, broadening the range of biological behaviors that can be probed at the mechanistic level (7, 8). One example of this expansion is the polymorphic fungus, *Aureobasidium pullulans*, which is emerging as a model for how the same cytoskeletal networks are repurposed to grow cells of remarkably different shapes and sizes (9–12).

One of the key tools that has enabled elucidation of actin organization is phalloidin, a phallotoxin produced by the death cap mushroom *Amanita phalloides* that binds with high specificity to F-actin and enables localization with high contrast and low background signal (13–15). However, for many fungi, phalloidin staining is ineffective (6, 16–18), and alternative approaches to visualizing actin do not provide comparable sensitivity or spatial resolution. Tools to enable live-cell imaging of actin organization involve fluorescent tagging of actin or actin-binding proteins. However, tagging actin directly compromises its function, and the use of tagged actin-binding proteins can result in poor decoration, and therefore, detection of subsets of actin networks (19). Thus, phalloidin remains the gold standard for visualizing cellular actin networks, and the failure of some fungal actins to bind phalloidin has hindered progress in those systems. Here, we find that a single residue change in actin (I75V) is shared among several fungi whose actin fails to bind phalloidin. Reversing that change was sufficient to allow phalloidin to bind *A. pullulans* and *A. nidulans* F-actin, with minimal to no detectable alteration to actin function. A similar approach would likely be successful in other fungal species.

## RESULTS

### Actin residue 75 is altered from isoleucine to valine in species where actin fails to bind phalloidin

**Fig 1:**
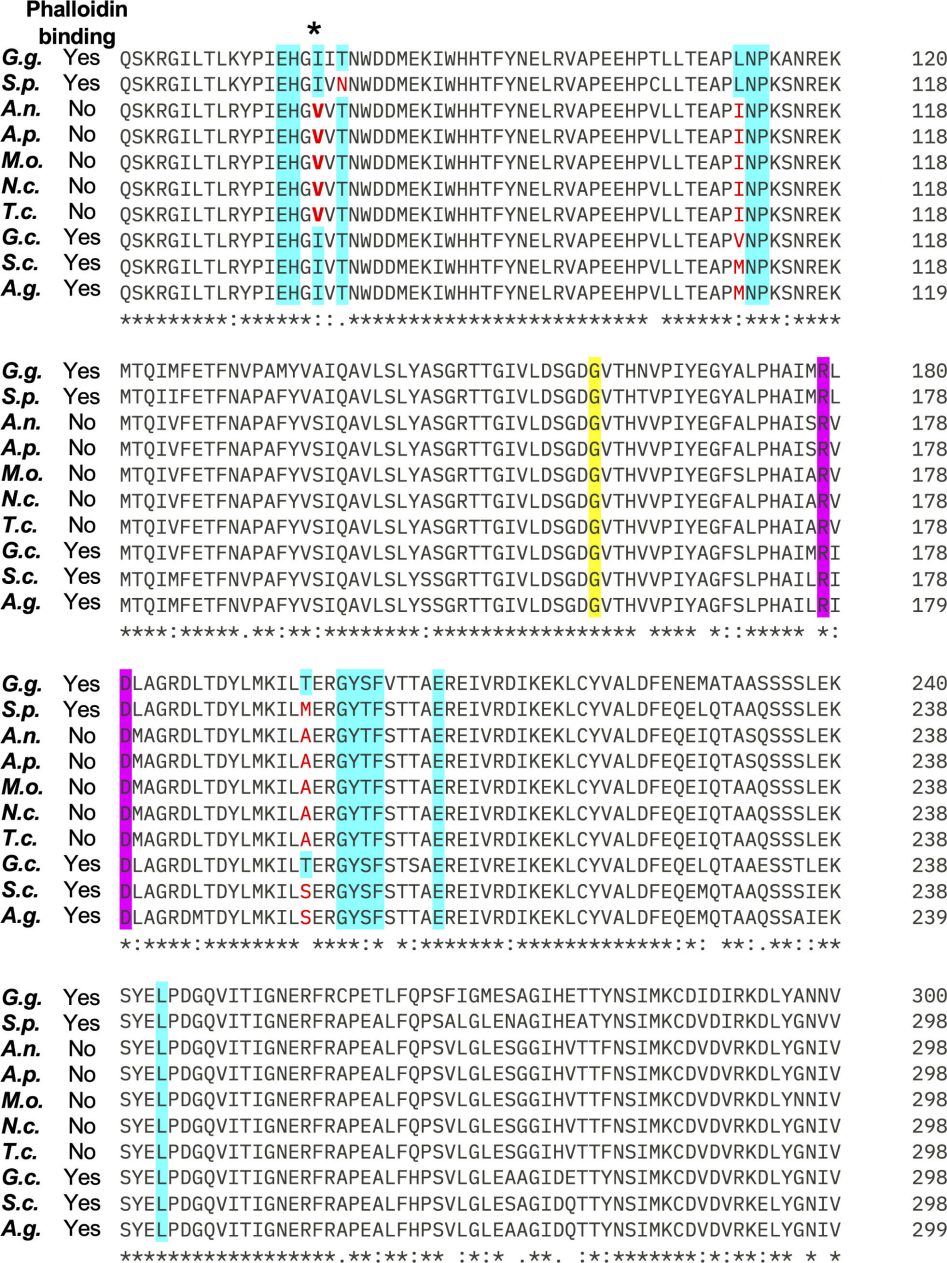
Actin sequence comparison and phalloidin staining. Sequences whose actin does (“Yes”) or does not (“No”) stain with phalloidin are indicated. The GenBank accession number for each sequence is in parenthesis: *Gallus gallus* (G.g., yes, NP_001385203.1), *Schizosaccharomyces pombe* (S.p., yes, NP_595618.1), *Aspergillus nidulans* (A.n., no, XP_664146.2), *Aureobasidium pullulans* (A.p., no, XP_029758358.1), *Magnaporthe oryzae* (M.o., no, XP_003719871.1), *Neurospora crassa* (N.c., no, XP_011394625.1), *Trichoderma citrinoviride* (T.c., no, XP_024751298.1), *Geotrichum candidum* (G.c., yes, KAF5095009.1), *Saccharomyces cerevisiae* (S.c., yes, NP_116614.1), and *Ashbya gossypii* (A.g., yes, NP_983171.1). Numbers in the figure indicate sequence position. Residues important for phalloidin binding based on structural data are highlighted in cyan, those implicated based on genetics in *S. cerevisiae* are highlighted in yellow, and those identified by both approaches are highlighted in magenta (23, 24). Cases where these residues are not conserved are in red. The isoleucine residue that is mutated to valine in all of the non-phalloidin-binding actin sequences is indicated with an asterisk. Sequence alignments were generated using Clustal Omega (26).

Phalloidin staining fails to label F-actin in several ascomycete fungi, including *A. nidulans*, *Magnaporthe oryzae*, *Neurospora crassa*, and *Trichoderma citrinoviride* (6, 16–18). These fungi all belong to the largest ascomycete subdivision, Pezizomycotina. However, F-actin is successfully detected by phalloidin in ascomycete species in Saccharomycotina (e.g., *Geotrichum candidum*, *Saccharomyces cerevisiae*, and *Ashbya gossypii*) as well as the more distant Taphrinomycotina (e.g., *S. pombe*) subdivisions (16, 20–22). This suggests that changes to actin resulting in failure to bind phalloidin may have occurred relatively recently in fungal evolution, perhaps being selected for toxin resistance in species that share a habitat with *A. phalloides*. To identify potential causal differences that impact phalloidin binding, we aligned each actin sequence and focused on residues shown to mediate phalloidin binding through structural and genetic studies (Fig. 1) (23, 24). We included *Gallus gallus* (chicken) actin because the electron cryomicroscopy structure of *G. gallus* actin bound to phalloidin was recently published (23). This structure showed that residues E72, H73, I75, T77, L110, N111, P112, R177, and D179 of one actin monomer (closer to the pointed end) and T194, G197, Y198, S199, F200, E205, and L242 of a second actin monomer (closer to the barbed end) in a filament interact with phalloidin (23). Many of the key residues that mediate phalloidin binding (e.g., I75, T77, L110, and T194) vary among fungi, but only residue 75 correlates with the ability to bind phalloidin. Residue 75 encodes isoleucine in species where actin binds phalloidin, and valine in species where it does not (Fig. 1). Isoleucine and valine only differ in a single additional methyl group on the side chain of isoleucine, but based on the actin-phalloidin structure (PDB id: 8v30), the CD carbon of isoleucine 75 (missing in valine) is very close (only 3.8 Å) to the center of the tryptophan ring in phalloidin. Because phalloidin binding to F-actin is highly cooperative (25), this could greatly weaken binding. We therefore reasoned that this single amino acid change could underlie the loss of actin binding to phalloidin.

### Scarless replacement of *ACT1* with *act1^V75I^* in *A. pullulans*

**Fig 2:**
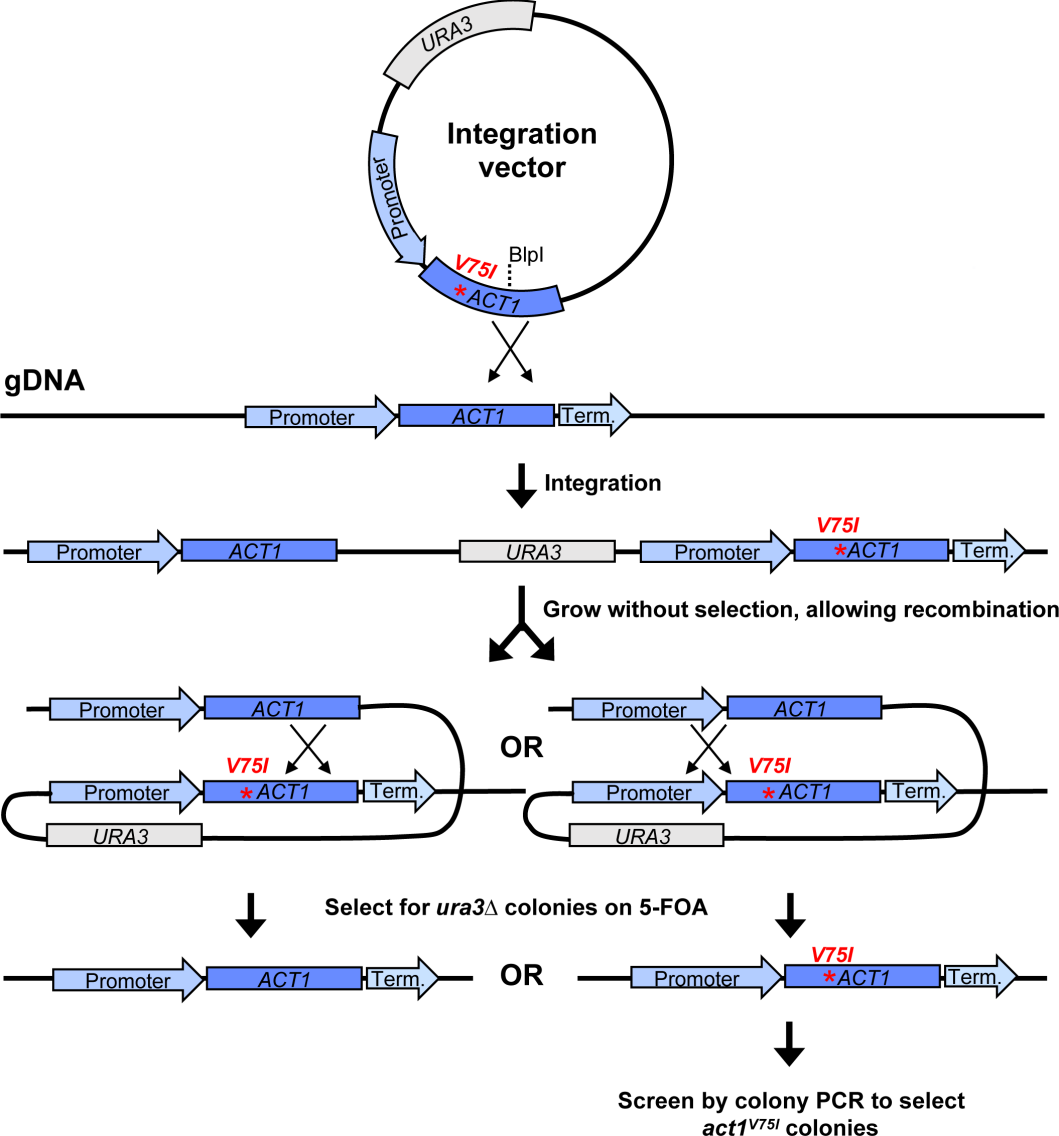
Scarless gene replacement strategy. Linearization of the integration vector with BlpI targets integration at the native *ACT1* locus. This results in the chromosomal *ACT1* and *act1^V75I^* sequences flanking *URA3* and the vector backbone. Colonies that have undergone homologous recombination to “pop-out” *URA3* are selected for on 5-FOA. Recombination downstream of the V75I point mutation pops out *act1^V75I^*, while recombination upstream of the V75I point mutation pops out *ACT1*, leaving a scarless *act1^V75I^* mutation. The *act1^V75I^* pop-outs are then identified by colony PCR.

**Fig 3:**
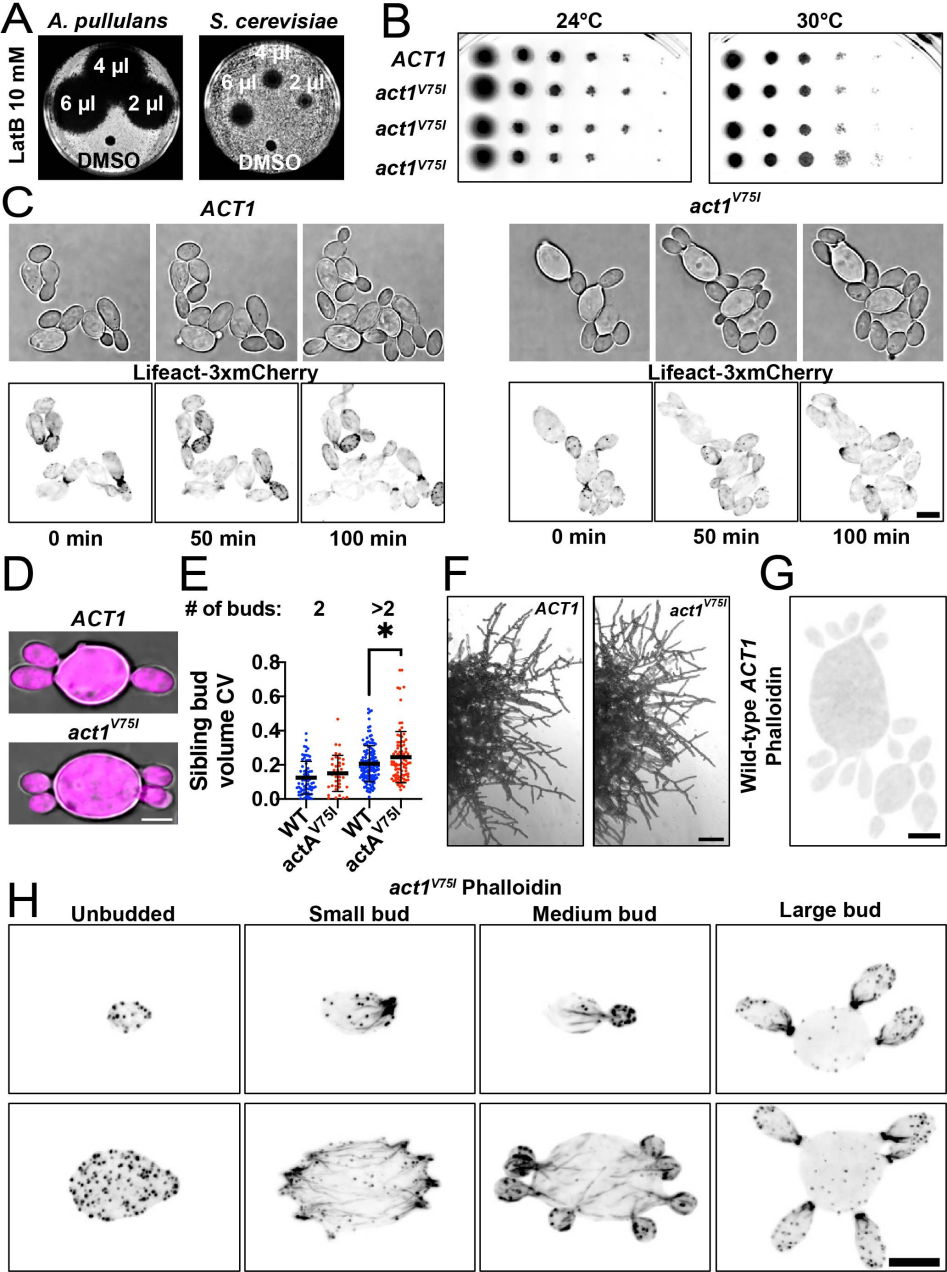
Phenotype of *act1*^V75I^ in *A. pullulans*. (**A**) Lawns of wild-type *A. pullulans* (DLY23540) or *S. cerevisiae* (DLY22370) cells spotted with the indicated amount of LatB (10 mM) or the carrier control (dimethyl sulfoxide [DMSO]). Dark areas (halo) indicate growth inhibition. (**B**) Growth of 10-fold serial dilutions of wild-type cultures (DLY23540) or cultures with the *act1*^V75I^ point mutation (DLY24813, DLY24814, and DLY24816) after 2 days of growth on YPD at the indicated temperatures. (**C**) Brightfield and maximum intensity projections of confocal images of live wild-type (DLY24628) and *act1*^V75I^ (DLY24872) cells expressing the F-actin marker Lifeact-3×mCherry. Scale bar, 5 µm. (**D**) Brightfield and maximum intensity projections of confocal images of wild-type (DLY24919) and *act1*^V75I^ (DLY25184) cells expressing 3×mCherry (cytoplasmic marker) used to measure bud volumes. Scale bar, 5 µm. (**E**) Sibling bud volume variability (CV = standard deviation/mean) of wild-type (DLY24919) and *act1*^V75I^ (DLY25184) mother cells growing the indicated number of buds (*n* = 71 wild-type and 36 *act1*^V75I^ mother cells with two buds, and 145 wild-type and 89 *act1*^V75I^ mother cells with more than two buds). Mean and standard deviation are indicated. Statistical significance calculated by two-way Student’s *t*-test (not shown, *P* > 0.05; *, *P* ≤ 0.05). (**F**) Images of 2-day-old colonies of *ACT1* (DLY23540) and *act1*^V75I^ (DLY24813) cells. Scale bar, 100 μm. (**G**) Maximum intensity projection image of a wild-type cell (DLY23540) fixed and stained with phalloidin showing no detection of F-actin networks. (**H**) Maximum intensity projection images of *act1*^V75I^ cells (DLY24813) with different sizes and bud numbers fixed and stained with phalloidin showing cortical patches and linear cables in small-budded and medium-budded cells. Large-budded cells show enrichment of F-actin at the neck. Scale bar, 5 µm.

Like other fungi in Pezizomycotina, *A. pullulans* has a valine at position 75 in actin, and our preliminary attempts to visualize F-actin with phalloidin staining in *A. pullulans* were unsuccessful (see Fig. 3E). To introduce a V75I point mutation at the native *ACT1* locus, we used a “pop-in/pop-out” strategy. This strategy takes advantage of efficient homologous recombination to first insert (“pop-in”) *act1^V75I^* next to endogenous *ACT1*, and then remove (“pop-out”) the wild-type *ACT1*, yielding a precise scarless gene replacement (Fig. 2). Insertion and removal are selected for using the counter-selectable marker *UR*A3 (27). *URA3* enables the synthesis of uracil, but it also converts the non-toxic compound 5-fluoroorotic acid (5-FOA) to the toxic metabolite 5-fluorouracil. Thus, cells with a wild-type *URA3* gene grow on media lacking uracil but die on media containing 5-FOA, while cells that lack *URA3* cannot grow on media lacking uracil but do grow on media containing 5-FOA (27).

We previously reported a *ura3*∆*HYG^R^* strain of *A. pullulans* (9). Into this strain, we transformed our plasmid with the *A. pullulans URA3* and *act1^V75I^* genes (Fig. 2). Digestion at the unique BlpI site was used to target integration of the plasmid to the *ACT1* locus (Fig. 2), and transformants were selected on media lacking uracil. Following integration, transformants have two copies of the actin gene, *ACT1* and *act1^V75I^*, separated by the *URA3* gene and plasmid backbone (Fig. 2). Growth on rich media allows rare recombination events to “pop-out” *URA3* (Fig. 2). Pop-outs were recovered by selection on media containing 5-FOA. When recombination occurs upstream of the V75I point mutation, a scarless *act1^V75I^* replacement is introduced at the native locus (Fig. 2). Successful *act1^V75I^* colonies were identified by colony PCR followed by sequencing.

### *act1^V75I^* does not compromise fungal growth or morphology in *A. pullulans*

As with other fungi, F-actin is necessary for the growth of *A. pullulans*, and depolymerization of F-actin with latrunculin B (LatB) blocks growth (Fig. 3A). In contrast, *act1^V75I^* mutant cells grow similarly to wild-type cells even at an elevated temperature, showing that the *act1^V75I^* allele functions to support cell growth (Fig. 3B).

*A. pullulans* exhibits yeast, hyphal, and meristematic growth morphologies (28, 29). In their yeast form, cells can be multinucleate and generate multiple buds in a single cell cycle (9, 11, 12). *act1^V75I^* mutants were morphologically similar to wild type. We recently showed that even mild perturbation of actin dramatically increases the number of mother cells growing buds of different sizes (30). In contrast, *act1^V75I^* mutants exhibited low bud volume variability (represented by CV = standard deviation/mean) (Fig. 3C through E), suggesting that actin networks in *act1^V75I^* mutants function normally. Additionally, *act1^V75I^* mutants retain the ability to grow hyphae that are morphologically similar to wild type (Fig. 3F). Although much weaker than phalloidin staining, actin networks can be visualized using fluorescent Lifeact (an F-actin-binding peptide) in *A. pullulans* (11). In an attempt to improve actin labeling, we tagged Lifeact with three tandem copies of mCherry codon-optimized for *A. pullulans*. This strategy works very well in *S. cerevisiae* (31) but was less effective in *A. pullulans* (Fig. 3C), highlighting the need to develop species-specific tools for F-actin detection. Regardless, Lifeact-decorated F-actin appeared similar in *act1^V75I^* mutants and wild-type cells (Fig. 3C). Together, these findings indicate that the V75I substitution does not appreciably affect actin function in *A. pullulans*.

### *act1^V75I^* allows for phalloidin staining of F-actin in *A. pullulans*

We next tested if *act1^V75I^* improved the ability to detect F-actin in *A. pullulans* using phalloidin staining. Formaldehyde fixation and staining of wild-type cells did not yield visible F-actin staining (Fig. 3G). However, when the same staining protocol was applied to *act1^V75I^* cells, phalloidin decorated F-actin networks similar in appearance to the dense Arp2/3-generated endocytic patches and linear formin-assembled actin cables seen in other fungi (Fig. 3H) (3). In large-budded cells, F-actin accumulated at the mother-bud necks, potentially forming an actomyosin ring for cytokinesis (Fig. 3H) (32). Thus, introducing a single residue change in *ACT1* is sufficient to enable phalloidin staining to detect F-actin in *A. pullulans*.

### *actA^V75I^* allows for phalloidin staining of F-actin in *A. nidulans*

**Fig 4:**
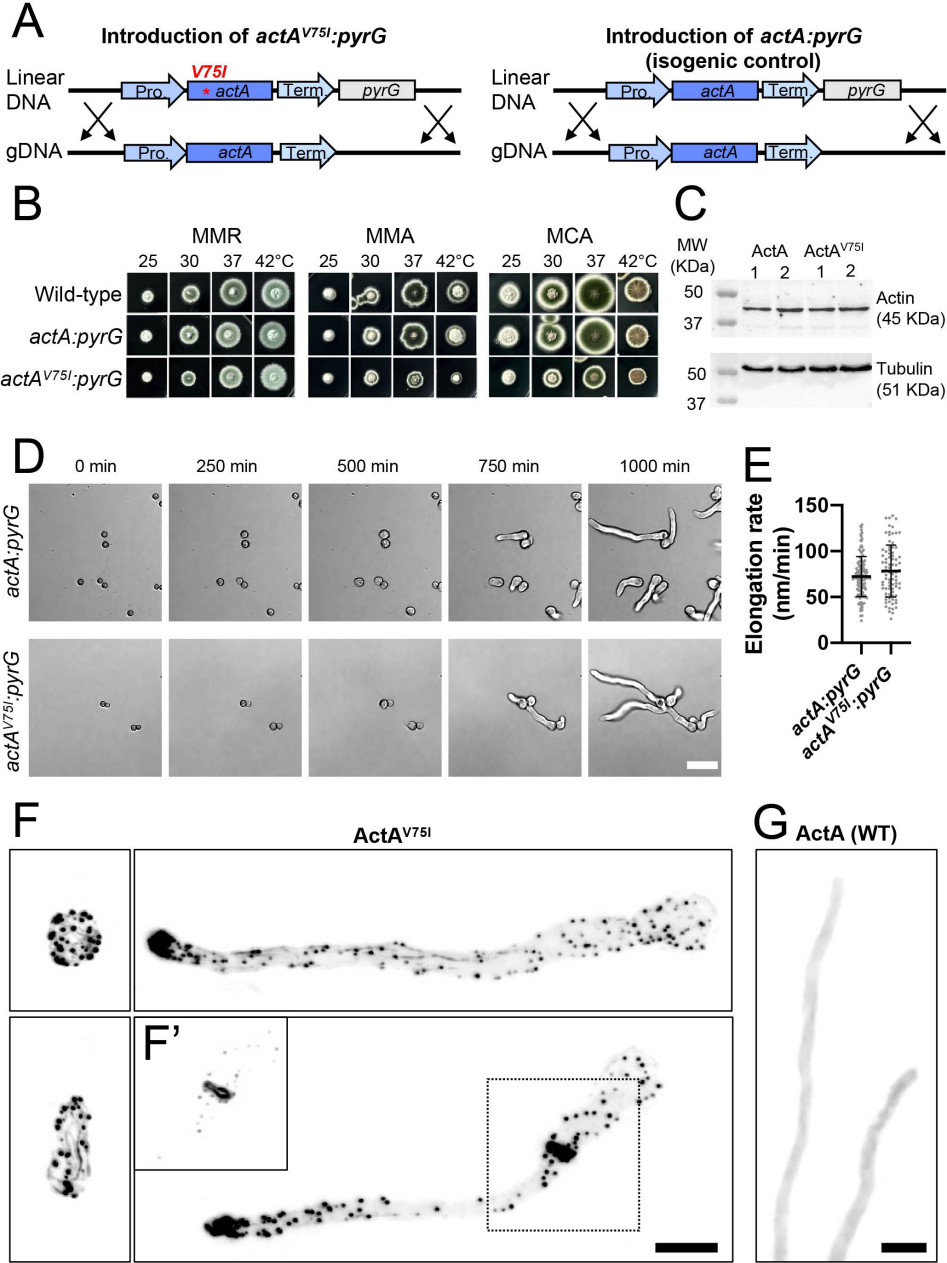
Phenotype of *actA^V75I^* in *A. nidulans*. (**A**) Schematic showing the integration strategy to introduce the V75I point mutation at the native locus linked to the pyrG selectable marker (*actA*^*V75I*^*:pyrG*) and the strategy used to generate the isogenic control (*actA:pyrG*). (**B**) Growth of a wild-type strain (DLY26757), the isogenic control strain, *actA:pyrG* (DLY26755), or the *actA^V75I^:pyrG* strain (DLY26756) after 3 days of growth on the indicated media at the indicated temperatures. (**C**) Western blot showing expression of ActA in two different clones of the isogenic control strain, *actA:pyrG* (ActA), and in two different clones of the mutant strain, *actA^V75I^:pyrG* (ActA^V75I^). (**D**) Widefield time-series of live *actA:pyrG* (DLY26755) and *act1*^*V75I*^*:pyrG* (DLY26756) conidia germinating on PDA at room temperature (20–22°C). Scale bar, 20 µm. (**E**) Elongation rates of *actA:pyrG* (DLY26755) and *act1*^*V75I*^*:pyrG* (DLY26756) germlings grown on PDA at room temperature (*n* = 132 *actA:pyrG*, *n* = 84 *actA^V75I^:pyrG* germlings). Statistical significance calculated by two-way Student’s *t*-test, *P* = 0.0793. (**F**) *act1^V75I^:pyrG* (DLY26756) conidia and germlings fixed and stained with phalloidin showing F-actin patches (puncta), cables (linear structures), and actomyosin ring (dashed box). The insert in F′ shows the area in the dashed box in F with the contrast adjusted to highlight the ring structure. Scale bar in F and F′ is 5 µm. (**G**) Phalloidin staining in *actA:pyrG* (DLY26755) germlings expressing wild-type actin shows no detectable F-actin structures. Scale bar, 5 µm.

The *A. nidulans* and *A. pullulans* actin protein sequences are identical. We therefore reasoned that the introduction of the V75I point mutation would similarly allow for the detection of F-actin by phalloidin staining in *A. nidulans*. The *actA^V75I^* mutation was introduced at the native locus using a construct with the *actA^V75I^* coding sequence and 3′ UTR followed by a selectable marker (*Aspergillus fumigatus pyrG*), allowing for selection of transformants on media lacking uracil (Fig. 4A). This approach maintains the native regulatory sequences flanking *actA*. To test if the introduction of *pyrG* downstream of *actA* altered *actA* function, a similar construct with wild-type *actA* was used to generate an isogenic control strain (Fig. 4A). Strains expressing *actA^V75I^:pyrG* and *actA:pyrG* grew comparably to wild type on synthetic complete medium supplemented with an osmotic stabilizer, 1 M sucrose (MMR), over a range of temperatures (Fig. 4B). However, on Cove’s complete (MCA) or synthetic complete media lacking an osmotic stabilizer (MMA), the *actA^V75I^:pyrG* strain had a slight growth defect (Fig. 4B). This growth defect was not due to reduced expression of the mutant ActA^V75I^ protein (Fig. 4C; Fig. S1). *actA^V75I^:pyrG* and *actA:pyrG* conidia both germinated and underwent polarized growth similarly (Fig. 4D and E). As these processes require a functional actin cytoskeleton (33, 34), ActA^V75I^ appears to be mostly functional, although readers interested in adapting this approach for use in other fungi should ensure that the V75I substitution does not disrupt processes of interest. ActA^V75I^ allowed for the detection of F-actin structures (actin cables, patches, and cytokinetic rings) by phalloidin staining in *A. nidulans* (Fig. 4F). Under these same conditions, phalloidin failed to detect F-actin in cells expressing wild-type ActA (Fig. 4G). Thus, substitution of actin Val75 by Ile suffices to enable F-actin detection by phalloidin in at least two fungal systems.

## DISCUSSION

We find that a phalloidin-interacting residue in actin, Ile75, is changed to Val75 in ascomycete fungi whose actin fails to bind phalloidin. Introducing a single residue change, V75I, is sufficient to enable phalloidin staining of F-actin in *A. pullulans* and *A. nidulans* and appears to perturb actin function only minimally (*A. nidulans*) or not at all (*A. pullulans*). This strategy to enable phalloidin staining will likely be applicable to additional ascomycete fungi. For example, *A. fumigatus* has an identical actin sequence to *A. pullulans*, and *S. cerevisiae ACT1* (which has an isoleucine at residue 75) supports the growth of *A. fumigatus* (17). In comparison, expression of fluorescently labeled F-actin-binding proteins has been shown to alter actin dynamics and disrupt function in multiple fungal systems (11, 35, 36). Additionally, F-actin-binding proteins can display preferences for specific cellular actin networks, making it difficult or impossible to detect all cellular networks (19, 37). Here, we present an additional tool for F-actin visualization that has the added benefit of evenly decorating all cellular F-actin networks with high signal over the cytosolic background (13–15, 19).

The function of fungal F-actin networks is very sensitive to actin expression levels (38–40). It is therefore important that the strategy used to introduce the V75I point mutation does not change actin expression. We used two strategies for introducing the V75I point mutation, both of which kept the native regulatory elements upstream and downstream of the actin coding sequence intact. If the regulatory elements are unknown, our genetic strategy for scarless editing can be used. This strategy relies on 5-FOA counterselection of *URA3+* cells, which has been shown to work in a wide range of fungi (27, 41–46). Precise gene replacement also relies on efficient homologous recombination, which is also found in many fungi. For species that preferentially use nonhomologous end joining, mutation of end joining factors has proven effective in increasing the frequency of homologous recombination (47–49). Thus, with minimal modifications, we hope that this strategy will enable phalloidin detection of F-actin in a wider range of fungi, expanding the tools available for investigating the roles of the actin cytoskeleton in diverse biological contexts.

## MATERIALS AND METHODS

### *A. pullulans* strains and maintenance

A complete list of strains used in this study is available in Table S1. All experiments conducted with *Aureobasidium pullulans* used strain EXF-150 and derivatives (50). For the LatB sensitivity assay, wild-type *S. cerevisiae*, YEF473 background (DLY22370), was used as a control (51). Unless otherwise indicated, *A. pullulans* was grown at 24°C in standard yeast extract, peptone, and dextrose (YPD) medium (2% glucose, 2% peptone, 1% yeast extract) with 2% BD Bacto agar (214050, VWR) in plates.

### Generation of *A. pullulans act1^V75I^* mutants

The *act1^V75I^* substitution (codon 75 GTC changed to ATC) was introduced using plasmid DLB4812. This plasmid has 1,212 bp upstream of the *ACT1* (protein ID: 347440) start codon and the mutant *act1^V75I^* coding sequence. The point mutation was introduced using primers. These sequences were introduced into the plasmid backbone pAP-U2-1 (Addgene ID# 236472) (10). This backbone includes the native *URA3* gene under the native URA3 promoter with the *scTEF1* terminator. Linearization with BlpI cuts in the *act1^V75I^* coding sequence, enabling integration (“pop-in”) at the native *ACT1* locus.

An additional selection step was used to “pop-out” the *URA3* cassette to generate the scarless *act1^V75I^* mutation. After transformation with linearized DLB4812 and selection of uracil prototrophs, transformants were grown overnight at 24°C in 5 mL YPD. These transformants have wild-type *ACT1* and the mutant *act1^v75I^* flanking the *URA3* cassette. To select for uracil auxotrophs that flipped out the *URA3* cassette, 10^6^ cells were plated on 5-FOA plates (6.71 g/L BD Difco Yeast Nitrogen Base without Amino Acids, BD291940, Fisher Scientific, 0.77 g/L Complete Supplement Mixture minus uracil, cat. no. 1004-100, Sunrise Science Products, 2% glucose, 50 mg/L uracil, 1 g/L 5-fluoroorotic acid, F10501-25.0, Research Products International, and 2% BD Bacto agar, 214050, VWR) and grown for 3 days at 24°C. Colonies growing on 5-FOA were checked by colony PCR to confirm introduction of the V75I point mutation using the Phire Plant Direct PCR Master Mix (F160S, Fisher Scientific), and the amplified *ACT1* (or *act1^V75I^*) coding sequence was checked by sequencing (Quintara Bio).

### Generation of *A. pullulans act1^V75I^* mutants with fluorescent reporters

To visualize F-actin with Lifeact, we used plasmid DLB4810. This plasmid contains the native *URA3* coding sequence and Lifeact-3×mCherry (three tandem copies of mCherry) driven by the *scACT1* promoter. This plasmid was built by using primers to introduce the Lifeact sequence into plasmid backbone pAP-U2-3 (Addgene ID# 236474) after removal of the *apACT1* promoter and 3×GFP sequences (10). Plasmid DLB4810 was designed to rescue uracil auxotrophy and integrate at the native *URA3* locus in an auxotrophic background, *ura3∆HYG^R^* (9). Restriction digest with ApaI and NruI results in a linear cassette flanked by homology to the native *URA3* locus. Transformants restore uracil prototrophy and insert Lifeact-3×mCherry at *URA3*.

All PCR fragments were amplified with Phusion Hot Start Flex 2× Master Mix (M0536L, NEB) following the manufacturer’s instructions, and the plasmids were built using NEBuilder HiFi DNA Assembly Master Mix (E2621L, New England Biolabs). Plasmids were confirmed by whole plasmid sequencing (Plasmidsaurus, Eugene, OR, USA).

To measure bud volumes, a similar approach was used to introduce the cytosolic marker, 3×mCherry, at the native *URA3* locus using the integration vector DLB4820, described previously (30).

### *A. pullulans* transformation

*A. pullulans* was transformed using PEG/LiAc/ssDNA as described previously (9). Briefly, cells were grown to a density of ~10^7^ cells/mL in YPD (4% glucose), harvested by centrifugation, and rinsed with sterile water followed by competence buffer (10 mM Tris, pH 8, 100 mM LiOAc, 1 mM EDTA, 1 M sorbitol). Cells were resuspended in competence buffer in a final concentration of 2 × 10^9^ cells/mL in a final volume of 50 µL (~10^8^ cells in each tube). At this stage, competent cells were stored in the −80°C freezer or used directly by adding 2.5 µL 40% glucose, 10 µL carrier DNA (10 mg/mL single-stranded fish-sperm DNA, 11467140001, Roche), 15 µL of transforming DNA (linearized plasmid ~1,000 ng/µL), and 600 µL of transformation buffer (10 mM Tris, pH 8, 100 mM LiOAc, 1 mM EDTA, 40% (wt/vol) PEG 3350). Cells were incubated in transformation buffer for 1 h at 24°C while rotating. Following the 1 h incubation, 30 µL 40% glucose was added, and the cells were heat shocked for 15 min at 37°C. Cells were centrifuged, and the transformation buffer was removed. For selection of prototrophs on media lacking uracil, cells were resuspended in 250 µL 1 M sorbitol and spread onto two drop-out uracil plates (6.71 g/L BD Difco Yeast Nitrogen Base without Amino Acids, BD291940, Fisher Scientific, 0.77 g/L Complete Supplement Mixture minus uracil, 1004-100, Sunrise Science Products, 2% glucose, and 2% BD Bacto agar, 214050, VWR).

### Generation of *A. nidulans act1^V75I^* mutants

A linear cassette containing 1,576 bp upstream of the ActA^V75I^ start codon, the *actA^V75I^* coding sequence, *actA* 3′ UTR (597 bp downstream of the stop codon), the *A. fumigatus pyrG* gene (selection marker), and a downstream homology region (1,456 bp after the 3′ UTR) was constructed by overlap extension PCR as described previously (52). The *actA^V75I^* coding sequence, 3′ UTR, and downstream homology region were amplified from genomic DNA, and the *pyrG* gene was amplified from 744 bp upstream of the start codon to 399 bp downstream of the stop codon from *A. fumigatus* gDNA, using Primestar polymerase (Takara). The V75I substitution was introduced using primers to replace codon 75, GTT, with ATT, and PCR fragments were designed to include overlapping homology regions. PCR fusion was carried out with the same polymerase, and successful fusion, and attainment of a single 7.3 kb linear fragment, was confirmed by gel electrophoresis and sequencing. A similar strategy was used to generate the linear cassette containing the wild-type *actA* coding sequence.

The cassettes were introduced into an *nkuA∆* background that is deficient for nonhomologous end joining, enabling improved homology-directed DNA insertion (48). The parent strain also carries the *pyrG89* mutation that confers uracil auxotrophic growth, enabling selection of positive transformants carrying the *A. fumigatus pyrG* gene on media lacking uracil. Transformants were obtained using standard protoplast-mediated transformation as described previously (53). Positive transformants were confirmed by diagnostic PCR using primers flanking the transforming DNA cassette, followed by sequencing to confirm the presence of the V75I missense mutation.

### Live-cell imaging of *A. pullulans*

For imaging experiments, a single colony was used to inoculate 5 mL of YPD (2% glucose). Cultures were grown overnight at 24°C to a density of 1–5 × 10^6^ cells/mL. Cells were pelleted at 9,391 rcf for 10 s and resuspended at a final density of ~7 × 10^7^ cells/mL. Approximately 2 × 10^5^ cells were mounted on an eight-well glass-bottomed chamber (80827, Ibidi) and covered with a 200 µL 5% agarose (97062-250, VWR) pad made with complete synthetic media (CSM; 6.71 g/L BD Difco Yeast Nitrogen Base without Amino Acids, BD291940, Fisher Scientific, 0.79 g/L Complete Supplement Mixture, 1001-010, Sunrise Science Products, and 2% glucose). All experiments were conducted at room temperature (20–22°C).

Growth of wild-type and *act1^V75I^* cells expressing Lifeact-3×mCherry was monitored by imaging on a Nikon Ti2E inverted microscope with a CSU-W1 spinning-disk head (Yokogawa), CFI60 Plan Apochromat Lambda D 60× Oil Immersion Objective (NA 1.42; Nikon Instruments), and a Hamamatsu ORCA Quest qCMOS camera controlled by NIS-Elements software (Nikon Instruments). Z-stacks (17 slices, 0.7 µm interval) were acquired using 50 ms exposure at 15% laser power (excitation 561 nm).

To measure bud volume variability, wild-type and *act1^V75I^* cells expressing 3×mCherry were imaged on the same confocal setup. The entire cell volume was acquired using 75 Z-slices at 0.2 µm step intervals. Exposure times of 50 ms at 50% laser power (excitation 561 nm) were used. The cytosolic marker 3×mCherry was used to segment and measure bud volumes. 3D segmentation and volume measurements were done in NIS-Elements General Analysis 3 software (GA3, Nikon Instruments).

### Live-cell imaging of *A. nidulans*

To grow *A. nidulans* strains for live-cell imaging experiments, cells were cultured on potato dextrose agar (PDA’ 39 g/L BD Difco potato dextrose agar, DF0014-17-6, Fisher Scientific) plates and incubated for 72 h at 37°C. Conidia were harvested from plates, resuspended in 1 mL of sterile water with 0.01% Tween 20, and stored at 4°C. For imaging, 10^7^ conidia were mounted on an eight-well glass-bottomed chamber (80827, Ibidi) and covered with a 200 µL PDA pad. Strains were imaged at room temperature (20–22°C) on a widefield Nikon ECLIPSE Ti2 inverted microscope with a Plan Apo 20× air objective (NA 0.75, Nikon Instruments), an sCMOS pco.edge camera (Excelitas Technologies), and an X-Cite XYLIS LED Illumination System (Excelitas Technologies) controlled by NIS-Elements software (Nikon Instruments). A single brightfield image was acquired every 5 min for 17 h to monitor conidia germination and elongation. Elongation rates were measured in FIJI by tracing the paths of elongating germ tubes, generating kymographs, and measuring the slopes from the kymographs (54).

### Imaging *A. pullulans* colony morphology

To determine if the *act1^V75I^* mutation alters colony morphology, *ACT1* and *act1^V75I^* colonies were used to inoculate 5 mL YPD and grown overnight at 24°C. The following day, 100–200 cells were plated on CSM plates supplemented with 10% (vol/vol) YPD and 7% agarose. Plates were grown for 2 days at 24°C. Colony edges were imaged at room temperature on an MSM 400 tetrad dissection microscope (Singer Instruments) equipped with PixeLINK PL-D752MU-T CMOS camera using a 4× objective (NA 0.10, Singer Instruments).

### Imaging phalloidin-stained F-actin

For *A. pullulans*, wild-type or *act1^V75I^* cells were grown in YPD as in live imaging experiments. Cells (500 µL) were fixed by adding formaldehyde (CAS # 50-00-0, Thermo Fisher) to a final concentration of 3.7% in YPD. Cells were fixed for 40 min at 24°C with agitation. Following fixation, cells were rinsed three times with phosphate buffered saline (PBS) and resuspended in 30 µL PBS with 0.1% Triton X-100. A stock solution of Alexa Fluor 488 phalloidin (A12379, Thermo Fisher) was prepared in anhydrous dimethyl sulfoxide (DMSO, 66 µM final concentration), and 1.5 µL was added to the 30 µL cells. Cells were incubated with the phalloidin at 24°C with agitation for 2 h in the dark, rinsed once with 100 µL PBS, mounted in SlowFade Glass Soft-Set Antifade Mountant (S36917, Thermo Fisher), and imaged immediately. We found that more rinses with PBS or allowing the samples to sit at room temperature for more than 1 h prior to imaging decreased the image quality. Fixed and stained cells were imaged on the same spinning disk confocal system used for live-cell imaging described above. The entire cell volume was acquired using 75 Z-slices (at 0.2 µm step intervals). Exposure times of 50 ms at 50% laser power (excitation 488 nm) were used.

For *A. nidulans*, conidia were collected from PDA plates and resuspended in sterile water with 0.01% Tween 20. Conidia were used to inoculate 1 mL of potato dextrose broth (no agar) in a 24-well plate and grown overnight at 24°C. The following day, germinated conidia were transferred to 1.5 mL Eppendorf tubes and fixed, stained, mounted, and imaged as described above for *A. pullulans*.

### Image processing

Confocal images were denoised using Denoise AI, and maximum intensity projections were generated in NIS Elements (Nikon Instruments).

### *A. pullulans* growth assay

To compare the cell growth of *A. pullulans* strains, a single colony was inoculated into 5 mL of YPD (2% glucose) and grown at 24°C for 48 h. Cultures were serially diluted five times in sterile deionized water in a 96-well plate and transferred onto solid YPD plates using a pin-frogger. Plates were grown for 48 h at 24°C and imaged on an Amersham Imager 680 (General Electric Company) using the colorimetric Epi-white settings.

To determine if depolymerization of F-actin disrupts growth of *A. pullulans*, wild-type *A. pullulans* and *S. cerevisiae* cells were inoculated and grown in YPD as described above. Cells (10^5^) from the overnight cultures were spread on YPD plates and briefly allowed to dry. The indicated amount of Latrunculin B stock (10 mM in DMSO) was spotted onto the plates along with a spot of DMSO as a negative control. Plates were grown for 2 days at 24°C and imaged as above.

### *A. nidulans* growth assay

To compare cell growth of *A. nidulans* strains, strains were grown at 37°C for 3 days on MCA plates (2% salts solution, 0.1% MCA trace elements, 1% vitamin solution, 1% casamino acids stock solution (15% [wt/vol]), 1% glucose (wt/vol), 0.2% peptone (wt/vol), and 0.1% yeast extract (wt/vol), 1% BD Bacto agar (214050, VWR) (wt/vol), pH 6.5. Salt solution was 26 g/L KCl, 26 g/L MgSO_4_·7H_2_O, and 76 g/L of KH_2_O_4_. Trace elements were 40 mg/L Na_2_B_4_O_7_·1H_2_O, 400 mg/L CuS0_4_·5H_2_O, 800 mg/L FePO_4_·2H_2_O, 8 g/L ZnCl_2_·7H_2_O, 800 mg/L NaMoO_4_, and 800 mg/L MnSO_4_·4H_2_O. Vitamin solution was 50 mg/L thiamine, 10 mg/L biotin, 100 mg/L nicotinic acid, 200 mg/L calcium D-pantothenate, 50 mg/L pyridoxine, 100 mg/L riboflavin, 100 mg/L para-aminobenzoic acid, and 24 g/L m-inositol) (55). After three days, conidia were harvested and resuspended in sterile water with 0.01% Tween 20.

For growth tests, strains were replicated using toothpicks onto MCA, MMA (5 mM ammonium tartrate, 10 g/L glucose, 20 mL/L salt solution, 1 mL/L trace elements, 1.5% BD Bacto agar [214050, VWR], pH 6.5, supplemented with nutritional requirements as appropriate), or MMR (MMA medium additionally supplemented with 1 M sucrose as osmotic stabilizer) plates. Plates were incubated at the indicated temperatures for 72 h and photographed with a Nikon D40 digital reflex camera.

### Western blotting

The levels of Act1 and Act1^V75I^ were determined by Western blotting. To grow mycelia for protein extraction, a 25 mL culture of Cove’s SC medium (MMA with appropriate supplements) was inoculated with ~1.5 × 10^7^ conidia/mL and grown for 16 h at 30°C. Mycelia were then collected by filtration through a Miracloth (Merck-Millipore), pressed dry, frozen, and lyophilized before proceeding with protein extraction.

Proteins were extracted from mycelia by grinding 6 mg of lyophilized mycelia with a ceramic bead (0.5 cm diameter) for 20 s in a FastPrep 24 (MP Biomedicals) at power level 4. Proteins were extracted from the powdered mycelia by the alkaline lysis method described previously (56), and 5% of the total extracts were loaded on 10% SDS-polyacrylamide gels. Gels were transferred to nitrocellulose membranes using the BioRad Mini-Protean Tetra Cell device (BioRad). Actin was detected with mouse anti-α-actin from Proteintech Europe (Manchester, UK) using a 1:500 dilution of the antibody, and α-tubulin (detected with mouse monoclonal anti-human TUBA4A from Sigma, using a 1:5,000 dilution) was used as a loading control. The peroxidase-conjugated secondary antibody was a goat anti-mouse antibody from Jackson Immunoresearch (Ely, UK) used at 1:1,000 for actin and 1:5,000 for tubulin. Blots were reacted with Bio-Rad Clarity Western ECL substrate and imaged using a Bio-Rad ChemiDoc imager. The uncropped raw Western blot is available in Fig. S1.

### Statistical analysis

All statistical analyses were done using GraphPad Prism. Data distributions were assumed to be normal, but this was not formally tested. Statistical comparison between indicated conditions was conducted using the two-sided Student’s *t*-test. Differences were considered significant if the *P* value was <0.05.

## Data Availability

The data generated in this study are available from the corresponding author upon reasonable request.

## SUPPLEMENTAL MATERIAL

Figure S1 msphere.00517-25-s0001.tiff Uncropped Western blots.

Table S1 msphere.00517-25-s0002.tiff Strains.

ASM does not own the copyrights to Supplemental Material that may be linked to, or accessed through, an article. The authors have granted ASM a non-exclusive, world-wide license to publish the Supplemental Material files. Please contact the corresponding author directly for reuse.
