# Supplementary figures and images for: A genetic strategy to allow detection of F-actin by phalloidin staining in diverse fungi
Source: mSphere. 2025 Sep 29;10(10):e00517-25. doi: 10.1128/msphere.00517-25 (PMC12570480; doi:10.1128/msphere.00517-25)

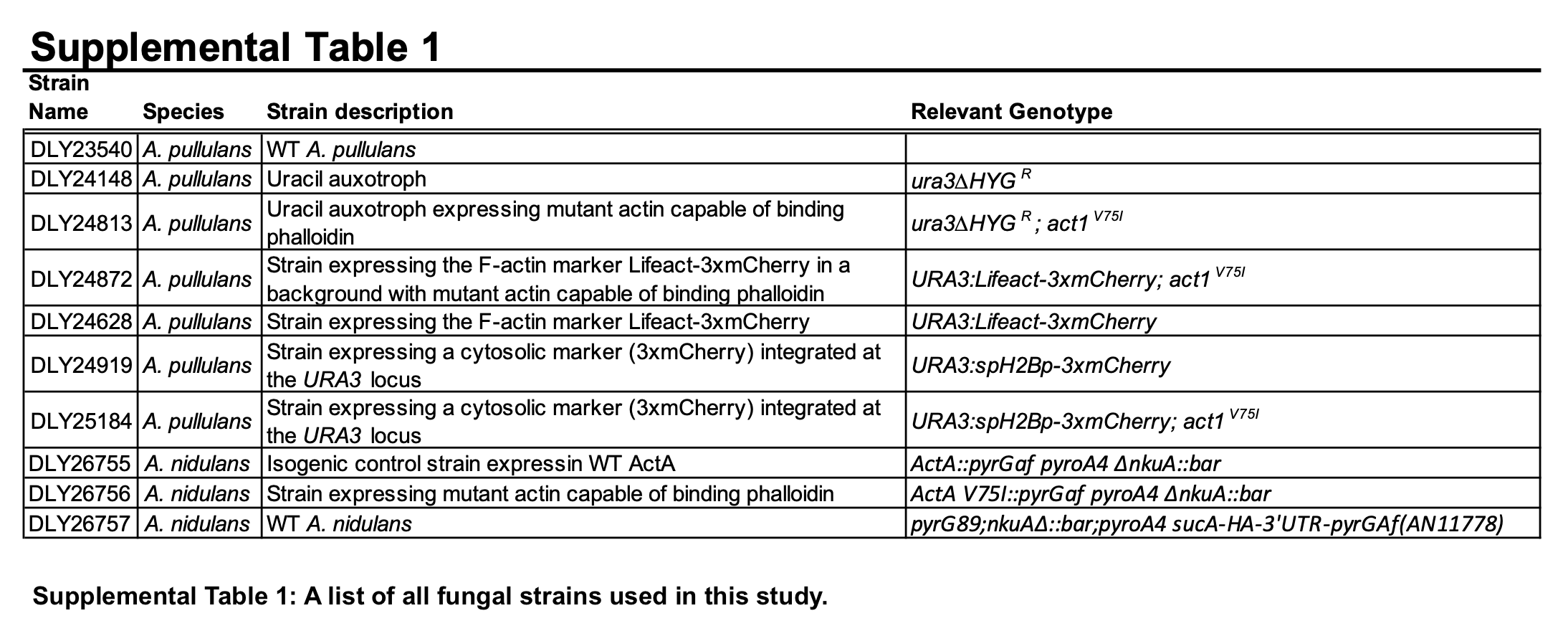

Supplement: Table S1 — Strains. [file msphere.00517-25-s0002.tiff]
